# Supplementary material for: Phosphomimicry on STAU1 Serine 20 Impairs STAU1 Posttranscriptional Functions and Induces Apoptosis in Human Transformed Cells
Source: Int J Mol Sci. 2022 Jul 1;23(13):7344. doi: 10.3390/ijms23137344 (PMC9266326; doi:10.3390/ijms23137344)
Supplement: Supplementary file 1 [file ijms-23-07344-s001.zip › Supp Table S2.pdf]

Supplementary Table S2

List of oligonucleotides used in this study
(mutated nucleotides are highlighted in yellow)

**Plasmids**	**Oligonucleotide primers**	
	Sense	Antisense
STAU1^{S20A}	5'-ctctcggatgcaggccacctataac-3'	5'-gttataggtggcctgcatccgagag-3'
STAU1^{S20D}	5'-ctctcggatgcaggacacctataac-3'	5'-gttataggtgtcctgcatccgagag-3'
STAU1^{T21A}	5'-ctcggatgcagtccgcctataactac-3'	5'-gtagttataggcggactgcatccgag-3'
STAU1^{T21D}	5'-ctcggatgcagtccgactataactac-3'	5'-gtagttatagtcggactgcatccgag-3'
STAU1^{Y22A}	5'-gatgcagtccaccgctaactacaacatg-3'	5'-catgttgtagttagcggtggactgcatc-3'
STAU1^{Y22D}	5'-gcagtccaccgataactacaac-3'	5'-gttgtagttatcggtggactgc-3'
STAU1^{Y24A}	5'-cagtccacctataacgccaacatgagag-3'	5'-ctctcatgttggcgttataggtggactg-3'
STAU1^{Y24D}	5'-gtccacctataacgacaacatgagag-3'	5'-ctctcatgttgctgttataggtggac-3'
